# Of Late Alarms, Long Queues, and Online Attendances: My Experiences of COVID Time

**DOI:** 10.1177/1077800420960157

**Published:** 2021-09

**Authors:** Sucharita Sarkar

**Affiliations:** 1 D.T.S.S. College of Commerce, Mumbai, India

**Keywords:** autoethnography, ethnographies, methodologies, pandemic, India, temporal

## Abstract

This essay has three autoethnographic, interconnected, temporal vignettes that narrate my lived pandemic experiences of mothering a teenage daughter, performing socially isolated housework, and teaching online classes. These personal experiences are located in specific Indian contexts through thick descriptions that accommodate more massive perspectives. Adapting Sarah Sharma’s concepts of power-chronography and temporal politics, I problematize my COVID-enforced slow time, and explore more deeply how my fraught COVID time experiences intersect with the multiple COVID times of others. I use this methodological format—autoethnography, thick description, and theorization—to make sense of my pandemic experiences at both microscopic and massive levels.

## Late Alarms and Hovering

*I wake up before the morning alarm—set at 8 a.m., much later than I used to—and immediately check the latest COVID stats on my phone. The numbers worry me, but seem rather distant from the cocoon of my lazy morning. I go to the window to look at the empty playground of the closed-down school next to our building, and I am greeted by the regular avian visitors—parrots, sparrows, crows, and pigeons—flying around, unaffected by the virus scare. I listen to birdcalls as I prepare breakfast for me and my 14-year-old daughter, readying her school stuff—laptop, pens, notebook, water bottle—on the table before waking her up.*

Lockdown was declared in Mumbai from March 21, 2020, with educational institutions and workplaces closing down indefinitely (Chaudhary & Joshi, 2020). The first thing I did was turn off my regular phone alarm, set at 5.20. a.m. for 6 days a week. Mumbai’s steep real-estate prices force most city colleges to start by 7 a.m., so that they can utilize the same space to accommodate multiple courses in morning, afternoon, and evening shifts. I usually leave home by 6.20 to reach my workplace and clock in my biometric attendance on time. When the lockdown started, the unexpected freedom from this routine surveillance was the most immediate effect I experienced. To me, the threat of the virus was still nebulous, the anxiety of infection still inchoate. For the first few weeks, I did not set any alarms, and my daughter and I got up whenever our bodies told us to. But when the lockdown extended indefinitely beyond the initial 21-day period, schools and colleges shifted unevenly and problematically to online teaching-learning. Our initial fluid, routine-free days have gradually coalesced into newer temporal structures that have shifted and messily redrawn the old boundaries separating work, home, and leisure. So, I reset the alarms, although at much later times than before. Late alarms mean that I can lie in bed for some more time, reading a story, before waking my daughter up. Late alarms mean less rush, and more time to listen to the rhythms of my night-owl body, and to my daughter.

The late alarm marks a new temporal regime of COVID time: a time of newer roles, rules, and expectations, located in a quasi-carceral, regulated space confined to the apartment where we stay and a range of shops in the immediate vicinity. My daughter’s school expects me to perform the role of a helicopter-mom: Their email advises that “close monitoring” by the parent “will help in maintaining cyber discipline.” My teenage daughter, however, protests against my initial invasive hovering. She claims that it betrays my lack of trust in her. So, I am learning to respect her space and yet hover discreetly from a mutually agreed distance. Beyond her monitored school hours, we have much more time to interact. We look up my old *Great Artists* books, watch sitcoms, listen to K-pop hits, bake cakes, and we talk. I go to another room when she video-chats with her friends, and she gives me space when I am writing. Outside of the school time-table’s insistence on helicopter parenting, we become closer, deeper mother–daughter together.

As the global pandemic disrupts our normative temporalities and enforces new routines or non-routines on us, maybe we can use our individual and shared COVID time/s to learn and inhabit new, slower, less-regulated schedules. This slow COVID time has different contours from the commodified “slow life” cultures so popular among the privileged. Sarah Sharma (2014) exposes how “slow life” cultures are maintained by the same broad nexus of neoliberal “temporal architectures” that impose fast-moving, speedup cultures of consumption (p. 111). My earlier college timetable emblematized speedup culture as it moved swiftly from one course to another from sunrise to night to accommodate more and more student-consumers. However, my present slow COVID time is based less on consumption and more on connection. It is also, because of the COVID disruption, necessarily outside institutional temporal control. This freedom is contingent and fleeting, because institutional routines are gradually being re-established, online now and offline later. So, I “hover” or linger in these liminal moments, experiencing the affirmations of my unmeasured, un-marketized, momentary COVID time.

But my story of late alarms is also a story of privileged, differential time which is inaccessible and meaningless to the vulnerable multitudes whose COVID times are marked by sudden viral illnesses, deaths, unemployment, and dislocation. The pandemic has jarringly halted what Sharma (2014) terms as global, neoliberal capitalism’s “discourse of speedup,” and has brutally interrupted our familiar experiences of time and space, but it has also aggravated and intensified the “temporal inequities” of speeded-up modernity (p. 138). Sharma theorizes “power-chronography” as a “way of locating how temporality operates as a key relation of power that manifests in the minutes, days, and lifetimes of different populations” (p. 137). I can reorganize my minutes and days into newer, partially freer temporal patterns. Outside my bubble of privileged COVID time, there are multitudes—like the jobless urban migrants waiting for trains to take them back to their rural hometowns—whose routines have been disintegrated beyond slowness into stoppage, as they have been abruptly pushed off the employer–employee relational grid that runs neoliberal power-chronography. I read about these migrants through the filters of online news websites.^1^ As I venture outside the isolated safe space of my apartment, rising leisurely from my late alarm, my path intersects with some of those who have been de-routined from the power-chronography of COVID-affected economies.

## Long Queues and Rotating Mops

*I wait in a queue outside the local Patel shop, along with around twenty others, standing in circles marked on the pavement at two-meter gaps. Two attendants stand at the shop entrance, one checking our temperatures, the other pouring sanitizer on our hands. They allow only five customers to go inside at a time, so the wait is long, but we are assured of maximized safety. As we wait, beggar children come and ask for money, or some ice-cream. They wear masks nowadays, like all of us do. After I buy what I need—which takes me over two hours, whereas before it took less than one—I go back home. My shiny, new, rotating mop awaits, because today I plan to sweep and mop the rooms. I have to do these chores now, as household helpers are no longer allowed inside residential buildings.*

Waiting in long queues, I have enough slow COVID time to observe the beggar children in thin, dirty, cloth masks that mimic my fancier, triple-layered safety mask. Yet, the precarity of their lives in unsanitary, crowded slums or unsafe pavements, and the necessity of being proximate to strangers for survival, make them extremely vulnerable to the virus. The simulation of safety in the beggar children’s masks actually masks the differences between “us” and “them.” We come into contact in brief moments and guarded spaces: Some of us buy food or give money to these children, careful not to touch them. The visible increase in beggars on Mumbai streets during lockdown may be traced to the sudden loss of employment of millions of unregulated migrant workers, including migrant child workers, in India (Ravichandran, 2020). In pre-COVID time, they have long been subjected to multiple “forms of temporal exploitation” (Sharma, 2014, p. 139). In COVID time, the government has officially announced measures for relieving migrant distress, which are, however, more oriented toward a post-COVID future time (Magazine & Sasi, 2020). But in present COVID time, I witness not just their exploitation, but their total expulsion from the safety nets of institutional temporal (and spatial) architectures. For those migrant workers who survive this prolonged expulsion and exclusion, what roles will they perform in the restored and relieved post-COVID temporal structures? Will it not be a return to the earlier forms of temporal exploitation, worsened by their experiences of falling out of present COVID time?

As for me, I have learned to perform other roles in slow COVID time: making food from end-to-end, cleaning the house by myself. In pre-COVID time, I paid my “maid” Aruna to do the housework: sweeping and mopping floors, washing utensils, making *rotis* (flatbread), and dicing vegetables. I could relax in the “slow space” of my home because of Aruna’s labor. “Slow spaces have their own biopolitical economy of time” and my slowness was generated by the exploited labor of Aruna (Sharma, 2014, p. 134). During lockdown, movement restrictions were imposed on domestic helpers for the first three months, which meant that Aruna did not come.

In Aruna’s absence, during my present slow COVID time, I learn the value of her labor that produces my slow space at home. Mimicking Aruna, I mopped the floors with a cloth mop for a few days, but then a flaring backache disabled me from bending waist down. This is when I purchased a fancy rotating mop from Amazon, so that I could mop standing up. The backache was aggravated by standing in long queues in front of shops. I resented the burden of housework, but I gained an embodied understanding of Aruna’s daily work routines. The only times I meet Aruna during lockdown embargo are at the beginning of each month: I meet her outside my building complex to pay her monthly salary: She tells me how some of her other employers have refused to pay her during the months of interrupted work. Aruna is a local Maharashtrian, not a migrant, and her plight is underreported and invisibilized by the media. Ironically, when Aruna re-joins work in July after lockdown is partially lifted, she rejects the rotating mop after using it once, saying that her arms ached, as if the new freedom from the abject bent-down posture was too heavy a sensation for her arms to carry. A return to old routines signals a shift from COVID time exclusions to pre-COVID temporal exploitations both for the migrant multitudes who are returning to their old cities and for local unregulated workers like Aruna who are returning to their old jobs, although available work openings have shrunk for both. COVID time has sharpened their struggles against exclusion. For privileged subjects like me, COVID time has been experienced through alterations, not exclusions.

## Online Attendances and Mandatory Lectures

*College campuses are closed, so I teach an online “Functional English” course twice a week. In each class on Google Meet, only around eight of the twenty-five enrolled students log in; on some days it is less. The absent students sometimes message me: They say their data-packs have gotten over as they cannot afford to buy new ones regularly. Many of their parents have lost their jobs. During class, I usually screen-share my presentation slides. So, I cannot see the students, whose cameras are switched off. When I ask them questions, only a few answer. I think, “Are the others even there? Or did they log in and go away, maybe to help their mothers with household chores.”*

I question the ways in which the English that I teach is “functional.” The sharp digital divides in India have generated intense debates about the forced shift to online education by educational institutions to manage their pandemic-disrupted academic calendars (Kundu, 2020). As a college faculty member, I am bound to take online lectures. It is “functional” for me in that it allows me to get my tangible salary and the intangible approval of my employers. I have to demonstrate its functionality by documenting the details of the classes I teach. But my functional COVID class time proves the non-functionality of those same classes to many of the students. They do not log in, or, even if they do, they do not listen or learn. Many of them are from poor, slum-dwelling, migrant families whose earning members have lost their jobs or who have shifted back to their rural homes (where the internet connection is poor). Those one-hour classes which ensure the continuity of my job and the comfort of my salary also, paradoxically, expose the opposite: How many of my students’ families are grappling with job discontinuities and internet discomforts.

However, the online hours I share with those eight students who do log in afford me glimpses into their familial contexts and personal anxieties, as they tell me stories of how their families have been affected by the lockdown, how they are worried about their immediate futures, whether they will be able to pay the fees for the next semester, whether they will be able to continue their education. My colleagues and I listen to their stories, and try and support them materially or emotionally wherever we can. In COVID time, the online teaching-learning hours imposed by an unprepared, inadequate educational system has deepened the socio-economic inequities among students, and many students are being excluded from the new online structures of college time maintenance.

Although I get frustrated at my present inability to connect to so many of my students, I also value this hourly connection with, and empathy for, those I can. I and my colleagues try to locate meaningful connections and empathies within these online hours—a mutual time to reach out, share, and support—even as we are coerced to deliver (or to simulate delivery of) the teaching-learning content expected of us. Sharma (2014) analyses the “out of time” experiences of “cab-lagged temporalities” of different classes of workers, like taxi-drivers (p. 78). Juxtaposed momentarily with their fast-traveling customers, they “exist in a differential and inequitable temporal relation with another group with whom they are expected to synch up” (Sharma, 2014, pp. 78–79). There is a palpable, differential and unequal disjuncture when I am juxtaposed with my students in the online classes, although the positions of privilege are reversed from Sharma’s case study. I am a privileged service-provider, while my students are poor customers. During the online class hours, the students are expected to log in and synch up with our times and timetables, but many of them fall off the synchronized grid of the online classroom. As employees with tenured jobs and secure incomes, we are shifting back into online “temporal architectures of time maintenance” which regulate our routines and expect our obedient participation (Sharma, 2014, p. 139). As we teach, take attendance, document and report our online teaching hours, our students slip in and out of these uneven, regulatory temporal structures, hovering between attendance and absence. Our relations are inequitable, but in those brief, unmonitored minutes located *within* or *outside* the regulated routines of online classes, we can communicate our concerns, and be “functional” for others in non-mandated, self-chosen, empathetic ways.

## Unlocking Lockdown Time

Sharma’s (2014) power-chronography advocates a temporal politics that “does not try to create more free time; it strives to free time from its bind” (p. 150). COVID time is unexpectedly making us experience time unbound and suspended from its normative bindings. I am experiencing and witnessing diverse granular experiences and massive awareness/es of present COVID time intersecting unevenly in offline and online transit spaces. At some of these intersections, I can enact a temporal politics to free COVID time from its bind. My daughter and I freely connect beyond her scrutinizing and binding school time, and share our COVID times in ways that respect each other’s boundaries, yet give us meaningful together-time. In my unbound COVID time, I learn the immense value of Aruna’s work. Even within my bound online class time, I try to free myself from my obligatory teaching role and be more empathetic and supportive. However, there are multiple intersections that exist at the borders of the bubble of my privileged COVID time, my real or virtual encounters with the beggar children or with migrants who have lost their jobs and homes, where I can only observe and record, but not enact either a temporal politics or an autoethnographic intervention.

In my unbound COVID time, I have more time to write. My reflexive writing is an embodied, experiential, and epistemic means of unpacking the clashing experiences and anxieties of COVID time. Ellis and Bochner (2000) state that, in reflexive autoethnographies “authors use their own experiences in the culture reflexively to bend back on self and look more deeply at self-other interactions” (p. 740). My COVID time autoethnography looks closely at my interactions with others: primarily my daughter, Aruna, my students; more peripherally with the beggar children and migrant workers. My hours of reflexive writing are an attempt to unlock and free my relational, differential COVID time from its bind/s, through a recalibration of routines, contemplation, interactions, empathy, and questionings.

## Notes

1. Although I briefly discuss about vulnerable subjects like urban migrants, child beggars, paid domestic workers, and students, this essay focuses primarily on my personal, microcosmic experiences of COVID time. It does not stand in for the COVID experiences of the most-affected groups in India.

## References

[bibr1-1077800420960157] Chaudhary A. Joshi A. (2020, March 20). Mumbai on partial lockdown as India tries to control virus. Bloomberg. https://www.bloomberg.com/news/articles/2020-03-20/mumbai-on-partial-lockdown-as-india-tries-to-contain-coronavirus

[bibr2-1077800420960157] Ellis C. Bochner A. P. (2000). Autoethnography, personal narrative, reflexivity. In Denzin N. K. Lincoln Y. S. (Eds.), Handbook of qualitative research (2nd ed., pp. 733–768). SAGE.

[bibr3-1077800420960157] Kundu P. (2020, May 5). Indian education can’t go online—Only 8% of homes with young members have computer with net link. Scroll.in. https://scroll.in/article/960939/indian-education-cant-go-online-only-8-of-homes-with-school-children-have-computer-with-net-link

[bibr4-1077800420960157] Magazine A. Sasi A. (2020, May 15). Covid economic package: Govt safety net for migrant workers and poor has little for now, more for later. The Indian Express. https://indianexpress.com/article/india/covid-economic-package-govt-safety-net-for-migrant-workers-and-poor-has-little-for-now-more-for-later-6410487/

[bibr5-1077800420960157] Ravichandran N. (2020, May 6). Where have the children on the streets gone? The Wire. https://thewire.in/society/covid-19-lockdown-children-beggars-workers

[bibr6-1077800420960157] Sharma S. (2014). In the meantime: Temporality and cultural politics. Duke University Press.

